# The mental health toll of COVID-19: significant increase in admissions to ICU for voluntary self-inflicted injuries after the beginning of the pandemic

**DOI:** 10.1186/s13033-023-00590-x

**Published:** 2023-07-15

**Authors:** Silvia Mongodi, Giulia Salve, Marta Ravasi, Damiano Rizzi, Matteo Mangiagalli, Valeria Musella, Catherine Klersy, Luca Ansaloni, Francesco Mojoli

**Affiliations:** 1 Anesthesia and Intensive Care 1St, Fondazione IRCCS Policlinico San Matteo, Pavia, Italy; 2 Department of Clinical-Surgical, Diagnostic and Pediatric Sciences, Università Di Pavia, Pavia, Italy; 3 Psychology department, Fondazione Soleterre, Milan, Italy; 4 Emergency Room, Department of Intensive Medicine, Fondazione IRCCS Policlinico San Matteo, Pavia, Italy; 5 Clinical Epidemiology and Biometrics, Fondazione IRCCS Policlinico San Matteo, Pavia, Italy; 6 General Surgery, Fondazione IRCCS Policlinico San Matteo, Pavia, Italy

**Keywords:** COVID-19, Self-harm, Suicide attempts, Self-inflicted injuries, Suicidal behaviours

## Abstract

**Background:**

COVID-19 outbreak deeply impacted on mental health, with high rate of psychological distress in healthcare professionals, patients and general population. Current literature on trauma showed no increase in ICU admissions for deliberate self-inflicted injuries in the first weeks after the beginning of COVID-19.

**Objectives:**

We tested the hypothesis that self-inflicted injuries/harms of any method requiring ICU admission increased in the year following COVID-19 outbreak.

**Methods:**

Retrospective cohort single-center study comparing admissions to ICU the year before and the year after the pandemic start. All patients admitted to polyvalent ICUs—Fondazione IRCCS Policlinico S. Matteo, Pavia, Italy from February 21st, 2019 to February 21st, 2020 (pre-COVID) and from February 22nd, 2020 to February 22nd, 2021 (post-COVID) were enrolled.

**Results:**

We enrolled 1038 pre-COVID and 854 post-COVID patients. In post-COVID, the incidence of self-inflicted injuries was 32/854 (3.8% [2.5–5.1]), higher than in pre-COVID (23/1038, 2.2%–p = 0.0014—relative increase 72.7%). The increase was more relevant when excluding COVID-19 patients (suicide attempts 32/697 (4.6% [3.0–6.2])–relative increase 109.1%; p < 0.0001).

Both in pre-COVID and post-COVID, the most frequent harm mean was poisoning [15 (65.2%) vs. 25 (78.1%), p = 0.182] and the analysed population was younger than general ICU population (p = 0.0015 and < 0.0001, respectively). The distribution of admissions for self-inflicted injuries was homogeneous in pre-COVID along the year. In post-COVID, no admissions were registered during the lockdown; an increase was observed in summer with pandemic curve at minimal levels.

**Conclusions:**

An increase in ICU admissions for self-inflicted injuries/harms was observed in the year following COVID-19 outbreak.

**Supplementary Information:**

The online version contains supplementary material available at 10.1186/s13033-023-00590-x.

## Background

Italy was hit by the new coronavirus-2019 disease (COVID-19) pandemic in February 2020, with deep impact on ICUs [1, 2] and healthcare systems [3]. To contain hospitals’ overload, stay-at-home orders and severe restrictive measures were introduced—called lockdown periods—limiting people movements and activities to reduce contagion. Beyond the impact on physical health, multiple warnings on the detrimental effects of COVID-19 pandemic on mental health have been raised: the patients admitted to ICU for COVID-19 showed an increased rate of post-traumatic acute stress disorders [4]; moreover, psychological distress was particularly high during COVID-19 in general population [5, 6] and in healthcare providers [7]. Limited data are available on the impact on admissions for self-inflicted injuries and harms [8–14]; as inferred from articles focusing on traumatic injuries, an overall decrease of admissions for trauma, including suicide attempts, is reported.

To test the hypothesis that an increase in voluntary self-inflicted injuries including any method of harm and requiring ICU admission was observed after COVID-19 outbreak, we compared the patients admitted to our ICUs the year before and the year after the beginning of the COVID-19 pandemic.

## Methods

### Population

All the patients admitted to polyvalent ICUs at Fondazione IRCCS Policlinico S. Matteo, Pavia, Italy were retrospectively enrolled. Patients were divided in pre-COVID (admission from February 21st, 2019 to February 21st, 2020) and post-COVID (from February 22nd, 2020 to February 22nd, 2021). We identified February 22nd as the beginning of COVID pandemic having been the first COVID-dedicated ICU in Italy and having admitted Italian patient N°1 in the night between February 21st and 22nd, 2020. ICU beds were 23 pre-COVID and expanded post-COVID to 35 beds where both COVID-19 and non-COVID-19 patients were admitted.

### Protocol

The study was approved by Comitato Etico Area di Pavia (20210084697/2021); written consent for data analysis was obtained. The primary endpoint was to detect an increase in ICU admissions for self-inflicted injuries/harms after the beginning of COVID-19 pandemic; we included any life-threatening method of voluntary self-inflicted injury or harm (*e.g.,* poisoning, hanging, defenestration, white weapon wounds, self-drowning…). Age, sex, method of harm, outcome (ICU length of stay and mortality) were recorded to compare pre- and post-COVID-19 populations. Social events as COVID-19 epidemiological data [15] were noted.

### Statistical analysis

For power analysis, being the incidence of admissions for self-inflicted injuries/harms highly dependent on each hospital’s catchment area, we considered as reference value for our ICU the one observed in pre-COVID population (2.2%); to identify an increase of at least 75% with power 80% and alpha error 0.05, 786 patients were required.

Primary endpoint: the proportion of patients was reported with its exact binomial 95% confidence interval; a single proportion test was used to compare suicidal attempts’ incidence in post-COVID period to the reference value.

Secondary endpoint: median and interquartile range (IQR) were used for quantitative variables, numbers and percentages for categorical ones. Normal distribution was assessed by Shapiro–Wilk test. Comparisons among categorical variables were evaluated with Pearson chi-square/Fisher’s exact tests; unpaired Wilcoxon/Mann–Whitney U-test were used for quantitative variables. All the analyses were conducted with STATA 14.2.

## Results

We enrolled 1892 patients, 1038 pre-COVID and 854 post-COVID. In pre-COVID, we identified 23 self-injured patients: 4 had a history of drug abuse, 10 had a psychiatric history (4 major depressive disorders of which 1 associated to personality disorder with previous self-injuries; 3 mixed anxiety and depressive disorders; 3 psychotic disorders of which 1 with history of self-injuries and 1 with borderline personality disorder). In post-COVID, we identified 32 self-injured patients: 7 had a history of drug abuse, 18 had a psychiatric history (8 mixed anxiety and depressive disorders of which 3 with history of self-injuries; 3 psychotic disorders of which 1 with history of self-injuries, 2 schizophrenias, 1 bipolar disorder, 1 binge eating disorder and Munchausen syndrome, 1 borderline personality disorder, 1 generalized anxiety disorder with panic attacks, 1 previous admission to psychiatric unit for unspecified reason). All self-injured patients were COVID-19 negative.

Post-COVID self-injured population was similar to pre-COVID in age, male gender prevalence, ICU length of stay and mortality (Table 1). Both in pre-COVID and in post-COVID, self-injured population was younger than general ICU population (p = 0.0015 and < 0.0001, respectively).

**Table 1:** Features of patients admitted to ICU in the 12 months before and after the beginning of COVID-19 pandemic

Data	Pre-COVID (n = 1038)	Post-COVID (n = 854)	P value
Age—years, median [IQR]	66.0 [51.0–77.0]	64.0 [52.0–73.0]	**0.0332**
Paediatrics—n (%)	48 (4.6)	38 (4.5)	0.912
Male sex—n (%)	643 (62.0)	557 (65.2)	0.148
Admission from—n (%):			
Emergency department	418 (40.3)	343 (40.2)	** < 0.001**
Extra-hospital medicine	5 (0.5)	4 (0.5)	
Surgical Unit	375 (36.2)	275 (32.2)	
Medical Unit	169 (16.3)	145 (17.0)	
Other ICU	41 (4.0)	80 (9.4)	
Rehabilitation centres	11 (1.1)	2 (0.2)	
Missing	18 (1.7)	5 (0.6)	
ICU length of stay—days, median [IQR]	4.0 [2.0–9.0]	4.0 [2.0–11.0]	0.6188
COVID-19 patients—n (%)	–	157 (18.4)	
Self-injured patients—n (%)	23 (2.2)	32 (3.8)	**0.049**
Age—years, median [IQR]	49.0 [26.0–65.0]^a^	36.0 [28.5–48.5]^a^	0.1124
Paediatrics—n (%)	1 (4.4)	2 (6.3)	0.759
Male gender—n (%)	13 (56.5)	17 (53.1)	0.803
ICU length of stay—days, median [IQR]	5.0 [3.0–13.0]	5.0 [2.5–9.0]	0.5033
ICU mortality—n (%)	4 (17.4)	2 (6.3)	0.191
Self-injury—n (%):			
Poisoning	15 (65.2)	25 (78.1)	0.182
Hanging	4 (17.4)	0 (0.0)	
Defenestration	3 (13.0)	3 (9.4)	
White weapon	1 (4.4)	2 (6.3)	
Drowning	0 (0.0)	1 (3.1)	
Other traumatism	0 (0.0)	1 (3.1)	

^a^Significantly lower than general ICU population of the same timeframe (pre-COVID: p = 0.0015; Post-COVID: p < 0.0001). P values in bold when statistically significant

*ICU* intensive care unit, *COVID-19* new coronavirus-2019 disease, *IQR* interquartile range

In post-COVID, the self-inflicted injuries/harms’ incidence was higher than in pre-COVID (32/854 (3.8% [2.5–5.1] vs. 23/1038, 2.2%; p = 0.0014), with a relative increase of 72.7%.

In both pre- and post-COVID, the most frequent harm method was poisoning (65.2 and 78.1%, respectively) (Additional file 1: Table S1). In pre-COVID, patients mainly used benzodiazepines (3, 20.0%) and alcohol 3 (3, 20.0%); 6/15 patients used multiple drugs. In post-COVID, the most frequent used drugs were opioids (8, 32.0%) and benzodiazepine (4, 16.0%); 10/25 used multiple drugs.

**Fig. 1:**
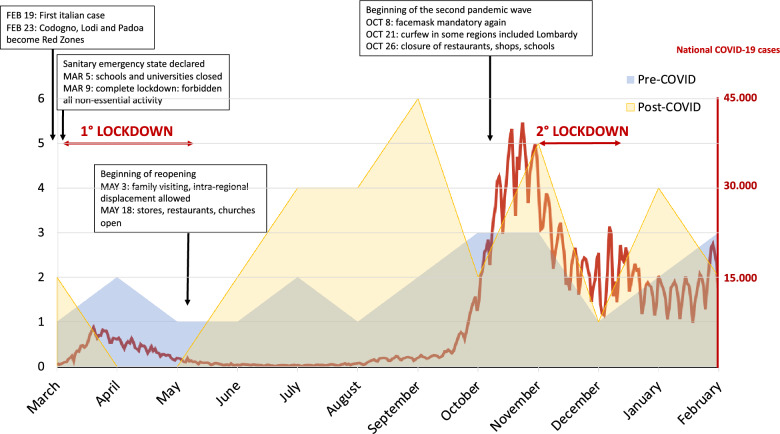
Admission of self-injured patients in ICU during the analysed year in pre-COVID (number of self-injuries/harms pre-COVID—blue wave—February 21st, 2019-February 21st, 2020) and post-COVID (number of self-injuries/harms post-COVID—yellow wave—February 22nd, 2020–February 22nd, 2021). Epidemiological data of COVID-19 positive cases in the corresponding timeframe of post-COVID is displayed in red. The main events in terms of closure and re-opening are marked. While a homogeneous distribution along the year of self-injuries/harms was observed pre-COVID, in post-COVID we registered no admissions during the first lockdown period (from March 9th to May 18th, 2020), where all non-essential activities were forbidden; the two admissions in March 2020th were registered before the beginning of the lockdown. A second shorter and less restrictive lockdown was established from November 3rd to December 5th, 2020th (also called red zone—maximal risk); afterward, Lombardy remained classified as orange (high risk)

The distribution of self-inflicted injuries/harms was homogeneous along the year in pre-COVID (Fig. 1). In post-COVID, no admissions were registered during the lockdown (from March 9^th^ to May 18^th^, 2020 – Fig. 1) while a peak was observed in summer, after the release of restrictive measures and with the Italian pandemic curve at minimal levels.

## Discussion

Our results showed an increase in ICU admissions for self-inflicted injuries or harms after the beginning of the COVID-19 outbreak.

This is the first study analysing a prolonged timeframe before and after COVID-19 outbreak and focusing on critically ill patients admitted to ICU for self-inflicted injuries, including any methods of harm.

Previous studies focused the attention on admissions for trauma during the lockdown, showing an overall reduction of trauma patients in the weeks following the introduction of stay-at-home orders [8–14]. Most of these studies report a reduction in road traffic accidents [8, 10, 12], as expected when restrictive measures limit population’s displacement, with an increase of penetrating trauma [8, 12, 14], of trauma related to do-it-yourself activities [9] and of drugs’ test positivity among trauma patients [10]. When specifically analysed, suicidal rate is reported as unchanged [8, 11]; Chiba et al. [10] report an increase of 38.5% of hospital admission for suicide attempts, although not reaching statistical significance in the observed population.

Most of these studies analyse a timeframe ranging from 6 weeks to 3 months [8–11, 13] after the beginning of the lockdown, a period where we also observed a limited number of admissions for suicide attempts. Moreover, these studies are focused on admission for trauma and do not include a frequent suicide method, as self-poisoning.

While most studies report no impact of COVID-19 on suicide-related thoughts and mortality rate in adults [16, 17], an increase in suicidal ideation and self-harm was observed in pediatric patients [18, 19].

The impact of COVID-19 pandemic on mental health could be due to the fear of an unknown and frequently severe disease, with perceived feeling of powerlessness, but also to the restrictive measures required to limit the spread of the disease, since also limiting social interactions, exasperating difficult domestic contexts, or potentially leading to unemployment [5, 6]. These effects are particularly evident on late adolescents, where sleep habits’ disruption and depression and anxiety symptoms have been described as frequent during the lockdowns [6]. Our population is however older than the one described in this previous study.

In our data, the rate of admission was higher when the lockdown was over, suggesting that the attention to public mental health by careful media communication, systematic screening [20] and follow up of fragile persons, should be kept high not only during but also after the peak of catastrophic events. This may be particularly true for young people, where a correlation between psychological distress and the use of smartphones or computers was observed, underlining the relevance of social media and information [4].

The absence of self-inflicted injuries/harms admitted during the lockdown may be explained by a less timely intervention due to higher isolation or to emergency system overload, as previously reported for cardiac arrests in our region [21].

Socio-economical factors may also play a role, since the impact of the lockdown on economic activities, with a reduction of national and international trades and an increase in cancelled enterprises in Lombardy [22] may have become a more significant burden after the first months of the pandemic.

Finally, during the COVID-19 waves, catchment areas for Lombardy hospitals significantly changed; our institution became a referral centre for COVID-19, with additional ICUs opened for COVID-19 patients only. We stopped being a referral centre for trauma from March 1st, 2020 to June 1st, 2020 and from November 1st, 2020 to the end on the analysed period (overall stop: 6 months and 20 days over 12 months). This represents a limitation of the study since it potentially led to an underestimation of the increase in self-harms/suicide attempts, since self-inflicted trauma (*e.g*., hanging, white weapon harms) could have been redirected by pre-hospital medical emergency teams to other trauma centres. This raises the potential interest in regional or national registries to correctly monitor this phenomenon.

Other limitations of the present study are that this is a single centre experience with comparison to only one year before COVID-19. A retrospective analysis with unadjusted comparison for age and sex was performed, data potentially affecting the incidence of self-injuries; however, pre- and post-COVID populations were similar in these general features.

## Conclusions

An increase in ICU admissions for self-inflicted injuries/harms was observed in the year following the beginning of COVID-19 outbreak; this suggests that long-term side-effects of the pandemic on the mental health of the general population are significant and may benefit of careful media communication, systematic screening and follow-up of fragile persons.

## Supplementary Information

**Additional file 1.**

## Data Availability

The datasets used and/or analysed during the current study are available from the corresponding author on reasonable request.

## Abbreviations

COVID-19: Novel-coronavirus disease
ICU: Intensive care unit
PEEP: Positive end-expiratory pressure
