# Supplementary material for: The mental health toll of COVID-19: significant increase in admissions to ICU for voluntary self-inflicted injuries after the beginning of the pandemic
Source: Int J Ment Health Syst. 2023 Jul 15;17:22. doi: 10.1186/s13033-023-00590-x (PMC10349498; doi:10.1186/s13033-023-00590-x)
Supplement: Supplementary file 1 — Additional file 1. [file 13033_2023_590_MOESM1_ESM.docx]

e-Table 1: Substances intake for voluntary poisoning in patients admitted to ICU in the 12 months before and after the beginning of COVID-19 pandemic

| **Substances – Pre-COVID (N = 15)** | **N** |
| --- | --- |
| Ethanol | 4 |
| Ethanol + Alprazolam | 1 |
| Benzodiazepine + morphine | 1 |
| Risperidone + Trihexyphenidyl + Valproate | 1 |
| Voluntary water-intoxication | 1 |
| Lithium | 1 |
| Quetiapine | 1 |
| Quetiapine + Sertraline | 1 |
| Escitalopram + Atenolol + Chlordiazepoxide | 1 |
| Paracetamol + Paracetamol/Codeine + Association of angiotensin-converting-enzyme inhibitors/calcium-channel blocker/diuretic | 1 |
| Unspecified | 2 |
| **Substances – Post-COVID (N = 25)** | **N** |
| Ethanol | 2 |
| Ethanol + Anti-histamine drugs | 1 |
| Ethanol + Alprazolam + Naproxen + Indomethacin | 1 |
| Flecainide | 1 |
| Bleach | 1 |
| Cocaine | 1 |
| Insuline | 1 |
| Methadone | 3 |
| Denaturated alcohol | 1 |
| Methadone + Diazepam | 1 |
| Methadone + Cocaine | 1 |
| Methadone + Cocaine + Quetiapine | 1 |
| Methadone + Cannabinoids + Opiods | 1 |
| Quetiapine + Bromazepam | 1 |
| Quetiapine + Olanzapine + Aripiprazole + Citalopram | 1 |
| Paracetamol + Pregabalin + Ibuprofen + Sulfamethoxazole/Trimethoprim + Amoxicillin/Clavulanate | 1 |
| Opioids | 1 |
| Unspecified | 1 |
| Clonazepam + Zolpidem + Levothyroxine | 1 |
| Paroxetine | 1 |
| Unspecified anti-epileptic drugs | 1 |
| Unspecified anti-psychotic drugs | 1 |

ICU: intensive care unit, COVID-19: new coronavirus-2019 disease.
